# Preoperative oral methadone for postoperative pain in patients undergoing cardiac surgery: A randomized double-blind placebo-controlled pilot

**DOI:** 10.1080/24740527.2019.1575695

**Published:** 2019-04-09

**Authors:** Timothy M. Bolton, Sarah O. Chomicki, William P. McKay, D. Ryan Pikaluk, Jeffrey G. Betcher, John C. Tsang

**Affiliations:** a Department of Anesthesiology, Perioperative Medicine and Pain Management, University of Saskatchewan, Royal University Hospital, Saskatoon, Saskatchewan, Canada; b Department of Anesthesiology, Perioperative Medicine and Pain Management, University of Saskatchewan, Regina, Saskatchewan, Canada; c Department of Anesthesiology, Regina Qu’Appelle Health Region, Regina, Saskatchewan, Canada; d Department of Critical Care, Regina Qu’Appelle Health Region, Regina, Saskatchewan, Canada; e Department of Cardiothoracic Surgery, Regina Qu’Appelle Health Region, Regina, Saskatchewan, Canada

**Keywords:** methadone, pain, postoperative, sternotomy, coronary artery bypass graft

## Abstract

**Background**: Inadequately controlled sternotomy pain after cardiac surgery can lead to delayed recovery and patient suffering. Preoperative intravenous methadone is effective for reducing both postoperative pain and opioid consumption. Despite ease of administration, the effects of preoperative oral methadone are not well described in the literature.

**Aims**: This pilot study investigated the effect of preoperative oral methadone on pain scores, analgesia requirements, and opioid-induced side effects.

**Methods**: A randomized double-blind placebo-controlled model was used with sampling of patients undergoing sternotomy for isolated coronary artery bypass graft (CABG) surgery (ClinicalTrials.gov registration no. NCT02774499). Participants were randomized to receive oral methadone (0.3 mg/kg) or oral placebo prior to entering the operating room. The primary outcome was pain scores on a 0–10 Verbal Rating Scale. Secondary outcomes included morphine requirements using patient-controlled analgesia (PCA), time to extubation, level of sedation, and side effects such as nausea, vomiting, pruritus, hypoventilation, and hypoxia over a 72-h monitoring time.

**Results**: Twenty-one patients completed the study. Oral methadone did not reduce pain scores in the methadone group (*P* = 0.08). However, postoperative morphine requirement during the first 24 h was reduced by a mean of 23 mg in the methadone group (mean difference, −23; 99% confidence interval [CI], 37–13 mg; *P* < 0.005). No reduction in pain scores or PCA morphine was observed beyond 24 h postoperatively. There was no difference in incidence of opioid-related side effects between groups throughout the postoperative period.

**Conclusions**: Though preoperative oral methadone did not reduce pain scores, morphine requirements were reduced in the first 24 h post-CABG.

## RÉSUMÉ

**Contexte**: Lorsqu’elle est mal contrôlée, la douleur secondaire à une sternotomie après une chirurgie cardiaque peut retarder le rétablissement du patient et lui occasionner de la souffrance. Laméthadone intraveineuse préopératoire est efficace pour réduire à la fois la douleur postopératoire et la consommation d’opiacés. Malgré que laméthadone orale soit facile à administrer, ses effets ne sont pas bien décrits dans la littérature.

**But**: Cette étude pilote portait sur l’effet de laméthadone orale préopératoire sur les scores de douleur, les besoins en analgésie et les effets secondaires induits par les opioïdes.

**Méthodes**: Un modèle randomisé à double insu contrôlé par placebo aété utilisé auprès d’un échantillon de patients subissant une sternotomie pour un pontage aortocoronarien unique (PAC) (NCT02774499). Les participants ont été randomisés pour recevoir de laméthadone orale (0,3mg/kg) ou un placebo oral avant d’entrer dans la salle d’opération. Le résultat principal était les scores de douleur obtenus sur une échelle d’évaluation verbale de 0 à 10. Les résultats secondaires comprenaient les besoins en morphine par le truchement d’une analgésie contrôlée par le patient (ACP), le temps écoulé avant l’extubation, le niveau de sédation et les effets secondaires tels que la nausée, les vomissements, le prurit, l’hypoventiliation et l’hypoxie pendant une période de surveillance de soixante-douze heures.

**Résultats**: Vingt-et-un patients ont participé à l’étude jusqu’à la fin. Laméthadone orale n’a pas diminué les scores de douleur chez le groupe avecméthadone (P = 0,08). Toutefois, les besoins en morphine pendant les premières 24 heures adiminué en moyenne de 23mg dans le groupe avecméthadone (différenc moyenne [IC 99%], −23 [37 à 13]mg; P< 0,005). Aucune diminution dans les scores de la douleur ou la morphine ACP n’a été observée au-delà des 24 heures postopératoires. Aucune différence n’a été observée entre les groupes en ce qui concerne l’incidence des effets secondaires liés aux opiacés tout au long de la période postopératoire.

**Conclusions**: Bien que laméthadone orale préopératoire n’ait pas réduit les scores de la douleur, les besoins en morphine ont diminué pendant les premières 24 heures suivant le pontage aortocoronarien.

## Introduction

Sternotomy is a painful part of all cardiac surgeries. Approximately 30% of patients will develop chronic, noncardiac pain poststernotomy, independent of the type of cardiac surgery.^1^ Uncontrolled acute pain is a risk factor for the development of chronic pain.^2^ A combination of preoperative and multimodal analgesia can significantly decrease acute pain.^3^ Recent research in spinal, obstetric, and cardiac surgery has shown the unique potential of intravenous methadone, which, in addition to its long half-life, has mu opiate as well as *N*-methyl-d-aspartate receptor (NMDA) effects on pain.^4^–^8^ No published studies to date have used methadone delivered via the oral route.

Moderate to severe postoperative pain is common in cardiac surgery patients, particularly during the first 48 h after surgery, even with potent opioids.^9^–^13^ Inadequately treated pain may have adverse physiologic consequences such as pulmonary dysfunction from pain-restricted coughing and breathing, sympathetic activation-induced myocardial ischemia, and arrhythmias.^14^

During early recovery, intravenous opioids are often administered for analgesia, either intermittently by nursing staff or by a patient-controlled analgesia (PCA) device.^15^ Repeated doses or boluses of analgesic medications result in fluctuating plasma opioid concentrations that can be avoided by the use of a single adequate dose of methadone (20–30 mg) that provides analgesia for approximately 24 to 36 h.^16^–^18^ An additional benefit of methadone is that it provides NMDA antagonism, which may play a role in chronic pain prevention, along with its known opioid effect as a mu agonist.^19^,^20^ This has been well described for intravenous methadone but not for oral methadone, which is convenient to administer preoperatively. The objective of this study was to generate pilot data to test this principle and learn whether oral administration will be efficacious before undertaking a larger study.

## Methods

### Trial design

We conducted a randomized blinded placebo-controlled parallel design pilot study with one-to-one allocation and allocation concealment. This trial was registered at ClinicalTrials.gov (NCT02774499).

### Participants

Included were patients undergoing sternotomy for elective, isolated coronary artery bypass graft (CABG) surgery. Participants were recruited by reviewing operating room schedules and were seen in the pre-assessment clinic (if outpatient) or on the wards (if inpatient) prior to surgery. If a patient was missed in the pre-assessment clinic, a researcher called the patient at home to explain the study in advance, to answer any questions, and to allow the patient time to consider his or her participation on the day of surgery. Signed informed consent was obtained.

Exclusion criteria included the following: <18 years of age, concomitant valve replacement, preoperative renal failure requiring dialysis or serum creatinine greater than 176 µmol/L, significant hepatic dysfunction (liver function tests more than twice the upper limit of normal), ejection fraction less than 30%, corrected QT interval (QTc) on ECG >440 ms for men and 450 ms for women. Many medications have interactions with methadone; though we did not exclude participants based on other medications they were taking, an already prolonged QTc was used for exclusion because of the known possibility of methadone further prolonging QT interval and subsequently inducing torsades de pointes.^21^ Other exclusion criteria included pulmonary disease necessitating home oxygen therapy, preoperative requirement for inotropic agents or intra-aortic balloon pump to maintain hemodynamic stability, emergency surgery, allergy to methadone, use of preoperative opioids, or recent history of opioid abuse. Opioid-tolerant patients were excluded according to the U.S. Food and Drug Administration definition: those taking at least oral morphine 60 mg daily or equianalgesic dose of another opioid for 1 week or longer.^22^

### Setting and location

The study was conducted in the Regina General Hospital in Regina, Saskatchewan, Canada. The trial received ethical approval from the research ethics boards of the University of Saskatchewan and the Regina Qu’Appelle Health Region and was conducted in compliance with good clinical practice.^23^

### Interventions

Participants received oral liquid methadone or an equal volume of sweetened syrup prepared as described below. This dose was consistently given when the patient was called to the operating room, just prior to transport. When used at doses of 0.2 to 0.3 mg/kg, intravenous methadone has not been associated with a higher incidence of opioid-related adverse events (compared with short-acting opioids).^4^,^16^ The conversion from parenteral to oral methadone has been reported variously from 1:0.7 to 1:2.^24^ For convenience and safety, our conversion ratio was 1:1.

### Anesthetic care

Patients received midazolam (2 mg) before being transported to the operating room. Standard Canadian Anesthesiologists' Society (CAS) monitors were applied, including a five-lead electrocardiogram and bispectral index monitoring. A radial arterial line and central venous catheter were inserted for invasive monitoring and access. Transesophageal echocardiography was used at the discretion of the anesthesiologist. Anesthesia was induced with midazolam (0–4 mg), sufentanil (0.5 μg/kg), propofol titrated to loss of lash reflex, and rocuronium (0.6–1 mg/kg). Sufentanil (0.5 μg/kg) was given as an infusion over 2 h. Additional sufentanil was given as clinically indicated, which was recorded and accounted for in the data analysis. Rocuronium was given to maintain paralysis and monitored with a nerve stimulator. Sevoflurane (0.4%–2.3%) and propofol (0–50 mcg/kg/min) were used to maintain anesthesia and were titrated to the bispectral index (values of 40–60) and to mean arterial pressures no less than 20% of baseline. Sevoflurane titration, nitroglycerin, phenylephrine, ephedrine, norepinephrine infusion, dobutamine, epinephrine, milrinone, or fluid boluses were used to control hemodynamics as clinically indicated. No steroids or antiemetic agents were given perioperatively. A propofol infusion (10–60 μg/kg/min) was initiated at sternal closure and maintained until the patient was transported to the intensive care unit (ICU).

### Postoperative care

As is normal ICU care at our institution, morphine (2–5 mg) intravenously up to every 5 min as needed was given for pain until extubated. Propofol was infused as per weaning protocol at a rate of 10–60 μg/kg/min for sedation until extubated. Once extubated, if analgesia was required, intravenous morphine (0.05 mg/kg) was given every 10 min until either the patient appeared to be resting comfortably or a maximum of five doses had been given. Once the patient’s base level of analgesia was established, he or she was provided with a PCA pump programmed to administer morphine (0.015 mg/kg), with a lockout interval of 6 min.

Gastrointestinal prophylaxis was maintained with ranitidine 50 mg intravenously (IV) every 8 h for 5 days, as well as pantoprazole 40 mg daily by mouth or IV, and was reassessed upon discharge from ICU. Acetaminophen 650 mg was given by mouth every 6 h while awake for 96 h and then 325–650 mg by mouth every 4 h as needed (not to exceed 4000 mg/24 h). Metoclopramide 10 mg by mouth or intravenously every 6 h as needed was ordered for nausea. Normal cardiovascular parameters were maintained using a combination of the following where clinically indicated: epinephrine, norepinephrine, dopamine, nitroprusside, milrinone, hydralazine, labetalol, and nitroglycerin. Fluid maintenance was achieved with Ringer’s lactate. Cardiopulmonary bypass was used in all cases. Patients were discharged from ICU and transferred to the Cardiac Surveillance Unit and then to the Cardiosciences ward once standard transfer criteria were met.

### Outcomes

Postoperative pain scores were measured and reported for 72 h postoperatively using a validated 0- to 10-point Verbal Rating Scale (VRS).^25^ Secondary outcomes included 24-h postoperative morphine requirements, time to extubation, level of sedation, and opioid-related side effects, specifically nausea, vomiting, pruritus, hypoventilation, and hypoxia during a 72-h monitoring period. The following baseline preoperative data were recorded: age, sex, weight, height, previous heart surgery, previous stroke, peripheral vascular disease, congestive heart failure, diabetes mellitus, lung disease, obstructive sleep apnea, chronic pain, hypertension, smoking, preoperative pain at rest and with cough, preoperative vital signs, and American Society of Anesthesiologists physical status classification. Total operating room time, total sufentanil, and total midazolam were also recorded.

The following postoperative data were collected: total time until extubation, total midazolam in ICU, total morphine given pre-PCA establishment, pain with cough postextubation, VRS pain scale (0–10) at rest and with cough, number of PCA requests, total morphine (mg), and number of incidents of nausea, vomiting, pruritus, constipation, urinary retention, hypoxia (SpO_2_ < 90% or needing supplemental oxygen), confusion/delirium as assessd by the validated Richmond Agitation–Sedation Scale (RASS), and hypoventilation (respiratory rate <8).^26^

### Sample size

A similar randomized controlled trail of intravenous methadone post–cardiac surgery reported a median VRS of 6 with coughing in the placebo group, with interquartile range 4 to 8, giving a mean deviation from the median of 2 points.^8^ From this it is possible to estimate a standard deviation of 1.48, allowing calculation of a sample size for parametric analysis.^27^ A sample size of 10 per group was calculated using a change in VRS with coughing of 2 points, an alpha level of 0.05, and power of 0.8.

### Randomization

A computer-generated simple randomization (www.random.org) was used with 1:1 allocation.

### Implementation

Randomization of patients was produced by our department research coordinator in another city; she had no further role in recruitment or assessment of outcomes. A sequentially numbered sealed envelope was opened the morning of surgery by a research assistant in the operating room holding area, stating whether the patient was randomly assigned to group A or B as well as the patient’s weight. Three bottles were used: bottles A, B, and C; bottles A and B were the methadone and placebo and bottle C was the sweetened syrup diluent. Both bottles A and B were dispensed as 10 mg/mL and diluted to a total volume of 5 mL in sugary syrup (bottle C) to mask any potential bitter taste. The 5-mL syringe containing either methadone plus diluent or placebo plus diluent was self-administered by the participant by mouth in the holding area prior to entering the operating room. The researchers were not present at the time of administration. A dose of 0.3 mg/kg was given (to a maximum of 30 mg).

### Blinding

The study design ensured concealment because recruiters and outcome assessors could not know the allocation of any participant. All of the researchers and members of the participants’ care team were blinded, including researchers, anesthesiologists, surgeons, and all other members of the operating room, ICU, and ward team.

### Statistical methods

We used a modified intent-to-treat analysis that removed participants who for any reason received no drug or placebo. The primary outcome, pain scores, and the secondary endpoint, total postoperative morphine consumption in the first 24 h as measured by total Patient-Controlled Analgesia (PCA) morphine, were assessed for normality of distribution by the Kolmogorov-Smirnov test and compared between groups with a *t* test if normally distributed and with a Mann-Whitney rank sum test if nonnormally distributed. The secondary outcomes with continuous data were similarly dealt with; secondary outcomes with categorical data were analyzed by chi-square test. Secondary outcomes were intended to have alpha levels corrected for multiple comparisons by the Sidak correction, but there were no significant differences in secondary outcomes. Numbers were insufficient for subgroup analysis.

## Results

### Recruitment

**Figure 1.**
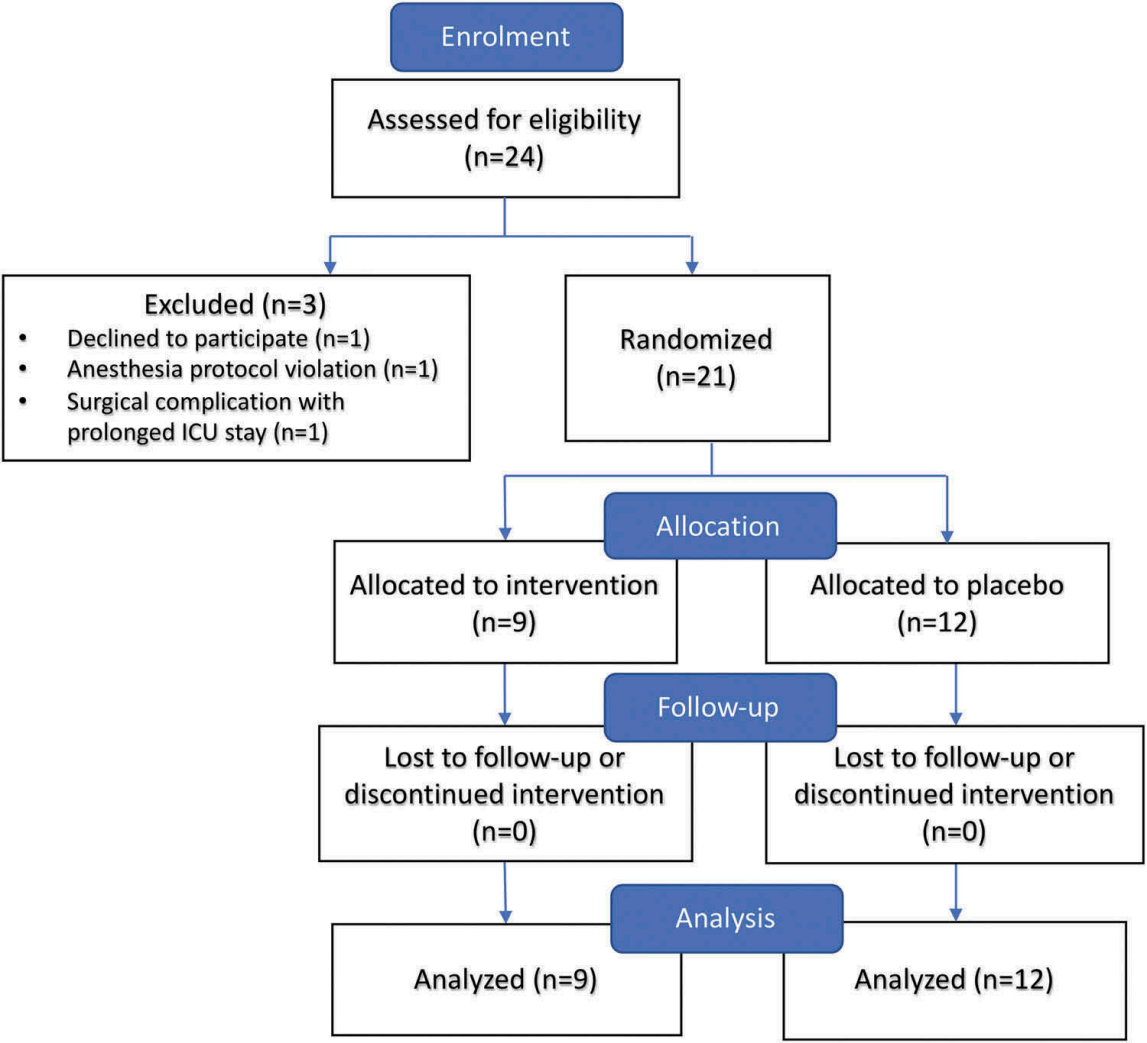
Patient allocation.

Recruitment was conducted from February to August 2016. Twenty-four patients consented to the study and were randomized. One patient was excluded because of significant intraoperative complications requiring prolonged ICU stay and abandonment of the protocol. One patient withdrew due to inability to tolerate the PCA. One patient was removed because of anesthesia protocol violation (high-dose ketamine). Twenty-one patients were included in a modified intent-to-treat analysis according to group allocation of either placebo or methadone. Twelve patients received placebo and nine received methadone (Figure 1).

### Numbers analyzed

Of 21 participants, 9 participants were analyzed in the intervention group and 12 were in the placebo group (Figure 1). Analysis was according to original assigned groups.

### Outcomes

**Figure 2.**
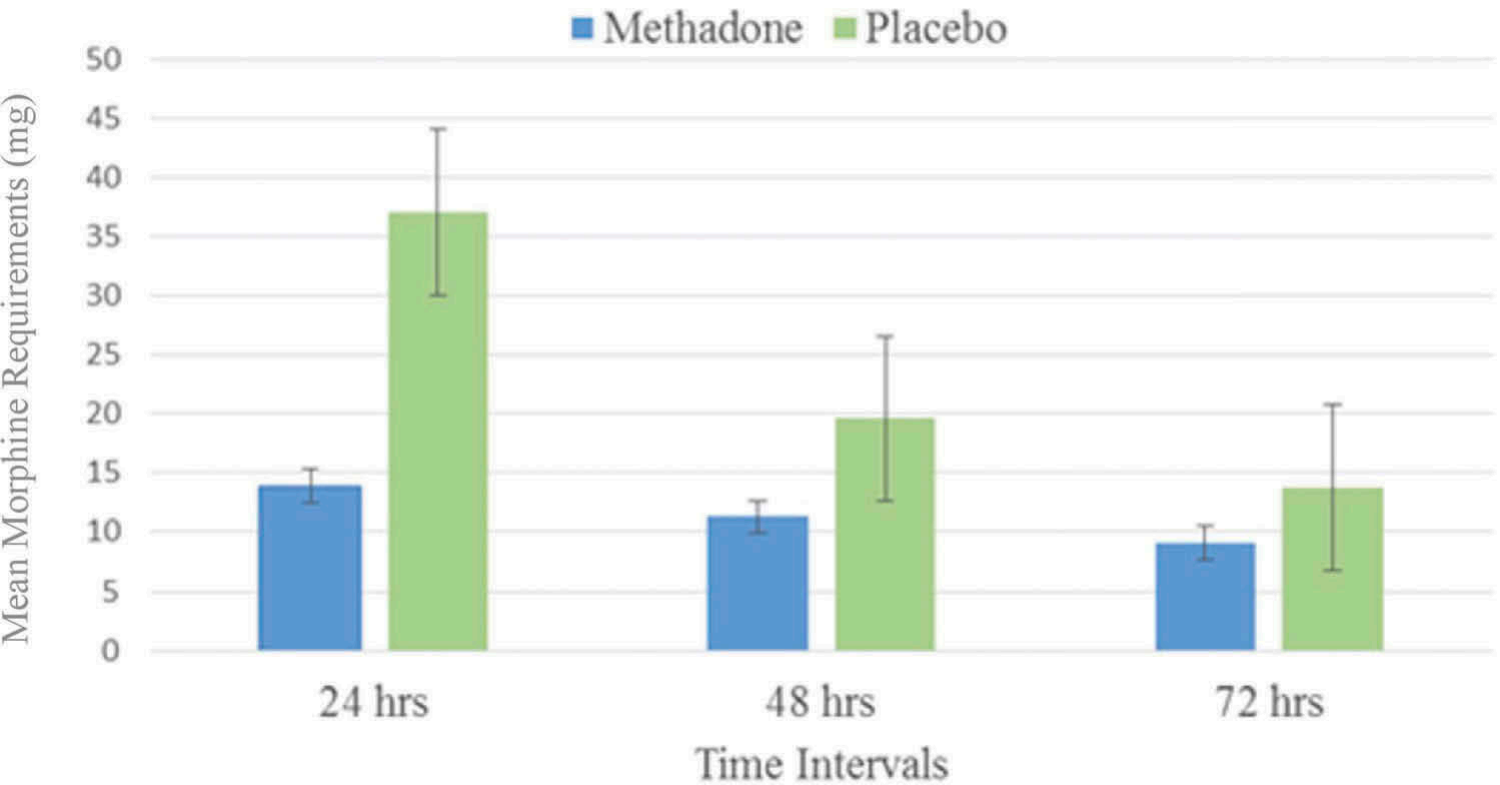
Postoperative PCA morphine. Morphine requirements via PCA pump in patients receiving either preoperative oral methadone or placebo. The difference was statistically significant in the first 24 h poststernotomy for coronary artery bypass graft (*P* = 0.003).

Baseline demographic and medical comorbidities were comparable between the groups (Table 1). However, two patients in the placebo arm had a history of chronic pain, whereas zero patients in the methadone arm reported the same. These two patients used 28.5 and 50.4 mg of PCA morphine in the first 24 h post-CABG, respectively, which appeared to fall within normal distribution, because the mean in the placebo group was 37.1 mg of IV morphine. Oral methadone did not reduce pain scores in the methadone group (*P* = 0.08; Table 2). However, postoperative morphine requirement during the first 24 h was reduced by a mean of 23 mg in the methadone group (mean difference, −23; 99% confidence interval [CI], 37–13 mg; *P* < 0.005; Figure 2). Nurse-administered morphine pre-PCA showed a statistically significant reduction between the methadone and placebo groups of 11.2 vs. 20 mg (mean difference, −8.8; 99% CI, 20.0–11.2; *P* = 0.007). No statistically significant difference in pain scores or PCA morphine was observed beyond 24 h postoperatively. The incidence of opioid-related side effects was not different between groups throughout the postoperative period (Table 2). Total intraoperative sufentanil, total operating room time, and postoperative midazolam were statistically equivalent. Mean volume of study substance ingested was equivalent, whether it was methadone or placebo (2.4 mL for a syringe containing methadone versus 2.6 mL for a syringe containing placebo; *P* = 0.09). Conversion between morphine and methadone is nuanced because of the differences between their respective pharmacokinetics and pharmacodynamics. Single-dose equianalgesic conversions of morphine to methadone have been reported from 1:1 to 14^28^; however, more recently, the mathematical relationship between methadone and morphine has been described as nonlinear, approaching a parabola^29^:

**Table 1.** Characteristics of the study population (*n* = 21).

	Methadone (*n* = 9)	Placebo (*n* = 12)
Age (years)	73	65
Weight (kg)	76	91
Previous heart surgery	2	2
Diabetes	2	4
Hypertension	4	7
Congestive heart failure	1	1
Stroke	0	2
Peripheral vascular disease	1	1
Obstructive sleep apnea	0	2
Smoking	3	5
Lung disease	0	1
Chronic pain	0	2

**Table 2.** Primary and secondary outcomes of methadone vs. placebo.^a^

	24 h		48 h		72 h	
	Methadone	Placebo	Methadone	Placebo	Methadone	Placebo
VRS at rest (0–10)	2.8	4.0	1.4	1.4	1.3	1.2
VRS with cough (0–10)	4.8	5.0	3.3	3.5	3.6	2.6
Time to extubation (min)	673	643				
RASS (+5 to −4)	−0.1	−0.1	0	0	0	0
Nausea	1.6	1.7	1.8	1.8	2.0	1.8
Vomiting	1.8	1.8	2.0	2.0	2.0	2.0
Pruritis	2.0	2.0	1.8	2.0	1.8	2.0
Constipation	2.0	2.0	2.0	2.0	1.9	1.9
Urinary retention	2.0	2.0	2.0	2.0	2.0	2.0
Hypoventilation (respiratory rate <8)	2.0	2.0	2.0	2.0	2.0	2.0
Hypoxia	1.3	1.3	1.3	1.5	1.6	1.7

VRS = Verbal Rating Scale for Pain; RASS = Richmond Agitation–Sedation Score.

^a^*P* > 0.05 for all secondary outcomes.

$y=\sqrt{ax}+b\;,where\;methadone\;(mg)\;\;\;\;\;\;\;\;\;\;\;\;\;\;\;\;\;\;\;\;\;\;\;\;\;\;=\sqrt{2.3xmorphine\left(mg\right)}+15$.

Using this mathematical relationship, at the mean dose of oral methadone given (24 mg), the conversion ratio approaches 1:1.5 and gives an oral morphine-equivalent dose of 35.2 mg. If we consider oral : parenteral morphine conversion to be 2:1 or 3:1, the equianalgesic dose given would be in the range of 11.7 to 17.6 mg of IV morphine, with a mean of 14 mg.

## Discussion

Though a single dose of preoperative oral methadone prior to CABG surgery did not reduce postoperative pain scores, it resulted in a significant reduction in morphine consumption in the first 24 h with similar rates of opioid-related side effects in both groups. This is the first prospective, randomized study using oral methadone in this population.

### Limitations

An obvious limitation of our study is its small size; despite this, the data showed a reduction of morphine consumption postoperatively. A second limitation is that high-risk patients and opioid-tolerant patients were excluded from enrollment. It is unclear what effect preoperative oral methadone would have in this group; however, one could speculate that it might show a similar benefit. Due to our small sample size, we only captured two patients with chronic pain and, due to randomization, they both received placebo; however, they did not use significantly more morphine than other patients in the placebo group and therefore did not skew the results. Extracorporeal circulation was used for every patient undergoing coronary artery bypass grafting. Although we know that initiation of cardiopulmonary bypass decreases the plasma concentration of other lipophilic opioids, such as fentanyl (by 53%) and sufentanil (by 34%), separation from cardiopulmonary bypass returns these sequestered narcotics into the systemic circulation of the patient^30^–^32^; however, formal pharmacokinetic studies of methadone for comparison are unpublished.

Psychological data such as history of depression, anxiety, and posttraumatic stress disorder were not collected on our patient population but could have been another factor contributing to our participants’ perceptions of their postoperative pain. The effect of methadone on long-term chronic pain development was not investigated in this study.

PCAs are not routinely used in our institution for postoperative pain control in sternotomy. We used PCA to measure morphine requirements as accurately as possible. Though our pilot study was underpowered to demonstrate superiority of methadone, equianalgesic conversion suggests that morphine was not merely replaced one-to-one by methadone.

### Generalizability

Our sample was made up of patients with symptomatic coronary artery disease requiring CABG. Though we excluded patients receiving valve replacements to maintain a homogeneous study population, we likely could have included these procedures without compromising homogeneity.

### Interpretation

Perioperative pain control is critical for good patient outcomes, because it enables early mobilization and may prevent chronic pain.^33^ Several analgesic regimens have been utilized for cardiac surgery, including neuraxial analgesia, local anesthetic infusion, and peripheral nerve blocks.^34^–^36^ However, many clinicians are reluctant to use these techniques because of their potential complications. Commonly, patients undergoing cardiac surgery receive either morphine or hydromorphone as well as nonopioid adjuncts for their analgesic requirements postoperatively. The usage of relatively short-acting agents such as morphine and hydromorphone results in plasma concentration fluctuations, which lead to fluctuating levels of analgesia. Administration of a long-acting agent such as methadone overcomes this pharmacodynamic limitation and provides a more prolonged and consistent baseline level of analgesia.

The literature offers little evidence regarding the utility of oral methadone; only recently have studies been published suggesting the utility of intravenous methadone.^4^,^5^,^8^ Our pilot study demonstrates that preoperative oral methadone may have some unexploited utility for patient analgesia. In Canada, oral methadone is readily available and is low cost. The ability to give methadone orally prior to transport to the operating theater has the practical benefits of being not only used in standardized preoperative order sets but also administered in concert with other agents to provide multimodal analgesia.

Possible mechanisms for our positive signal likely include a combination of methadone’s long duration of action (24–36 h) as well as its dual mu agonist and NMDA antagonist effect.^17^,^18^ The prolonged duration of action of methadone provides analgesia until the patient is extubated and potentially even discharged from the ICU. The involvement of NMDA receptors has been shown to be useful for postoperative analgesia in studies of ketamine.^37^

Our study was not adequately powered to show that preoperative oral methadone is superior to placebo for reducing postoperative morphine consumption; however, a larger trial should be carried out to further elucidate the clinical significance of this result.

Currently there are only a small number of studies that have investigated the utility of perioperative intravenous methadone. Our study is unique in its route of administration, which has obvious clinical implications. Oral methadone could be given easily in the preoperative setting for many surgeries, although further research is required, including a larger multicenter trial, investigation of other surgical types, direct comparison of oral versus intravenous methadone, and impacts on the incidence of chronic pain following surgery. In summary, our pilot study suggests that a single administration of preoperative oral methadone prior to sternotomy results in equianalgesic reduction in morphine consumption in the first 24 h with equivalent rates of opioid-related side effects compared to placebo.
